# Understanding tuberculosis transmission and progression: A prospective cohort study of index cases and close contacts in Moldova

**DOI:** 10.1371/journal.pone.0313270

**Published:** 2024-12-05

**Authors:** Rehan R. Syed, Donald G. Catanzaro, Naomi Hillery, Valeriu Crudu, Elena Tudor, Nelly Ciobanu, Alexandru Codreanu, Maryam Kheirandish Borujeni, Antonino Catanzaro, Timothy C. Rodwell

**Affiliations:** 1 Division of Infectious Diseases and Global Public Health, Department of Medicine, University of California San Diego, La Jolla, California, United States of America; 2 Department of Biological Sciences, University of Arkansas, Fayetteville, AR, United States of America; 3 The Herbert Wertheim School of Public Health and Human Longevity Science, University of California San Diego, La Jolla, California, United States of America; 4 The Institute of Phthisiopneumology “Chiril Draganiuc,” Chisinau, Republic of Moldova; 5 Department of Industrial Engineering, University of Arkansas, Fayetteville, Arkansas, United States of America; 6 Division of Pulmonary, Critical Care and Sleep Medicine, Department of Medicine, University of California San Diego, La Jolla, CA, United States of America; Universiti Kuala Lumpur, MALAYSIA

## Abstract

**Objectives:**

This study aims to determine the progression rate, risk factors and timeline for the progression from exposure to active tuberculosis (TB) in a high-risk population. Using a prospective cohort in the Republic of Moldova, we investigated pulmonary TB disease progression among close contacts of patients with TB in a low-burden country with high rates of drug-resistant TB.

**Methods:**

Close contacts of patients with newly diagnosed TB were recruited and monitored to evaluate for progression rates to active TB. Data collected included demographic information, medical and exposure history, and clinical samples. Follow-up clinical evaluations of close contacts were conducted at regular intervals over at least 24 months to monitor for progression to TB disease.

**Results:**

The overall incidence rate of TB disease among close contacts was 3.7%. Among the close contacts, 2.3% were identified as progressor cases, developing TB disease more than 30 days after index case treatment initiation. Thirteen (1.3%) were co-prevalent cases, diagnosed within 30 days of index case treatment initiation. Identified risk factors for progression included male sex, active tobacco use, prior TB infection, and frequent, prolonged exposure to index cases. Close contacts with daily exposure of more than eight hours had a significantly higher risk of disease progression (adjusted OR: 4.28, 95% CI: 1.79–10.23).

**Conclusion:**

The incidence of TB disease among close contacts was consistent with global findings, highlighting the need for enhanced diagnostic tools and targeted interventions to manage TB transmission and progression. These results underscore the importance of contact tracing and progression monitoring in low-burden, high drug-resistant TB settings. Future research should focus on developing a better understanding of factors contributing to the risk for and timeline of TB disease progression, and more precise methods, including biomarkers, to identify individuals at the highest risk for progression from TB exposure to active disease.

## Introduction

Tuberculosis (TB) remains a formidable global public health challenge, with an estimated 10.6 million people falling ill and 1.3 million people dying from TB worldwide in 2022 [1]. While progress has been made in prevention, case identification, and treatment, TB remained the second leading cause of death globally from a single infectious agent in 2022, after COVID-19, and progress remains short of the WHO End TB Strategy: 2025 milestones targets. As observed in 2020 and 2021 during the COVID-19 pandemic, a period in which TB case identification fell due to disruptions in health services globally, the identification of TB cases is key to enrolling patients in care and employing public health measures to prevent the propagation of the pathogen *Mycobacterium tuberculosis*.

Despite extensive experience with the disease, there remain significant gaps in the understanding of TB infectious pathophysiology, transmission dynamics, and progression risks profiling among those exposed. The process of TB infection and progression remains opaque, and recent proposals have suggested that the framework of the fundamental TB infectious states should be redefined [2]. Furthermore, while various screening protocols may be employed based on community and individual risk factors, medical and public health workers frequently rely upon index case identification and close contact tracing to treat and prevent the spread of TB. Therefore, an improved understanding of the process of TB transmission and progression to disease, particularly in settings with varying TB prevalence, could help identify individuals at higher risk for infection, progression to active disease, and potential for further transmission to other individuals within their social network.

Multiple studies have attempted to address these gaps in the literature, with recent efforts focusing on better characterizing TB infection and disease incidence among exposed individuals. *Whalen et al*. noted a secondary attack rate (SAR) for TB disease of 3.0% and a SAR for TB infection of 47% in the high-prevalence setting of Kampala, Uganda [3]. A systematic review and meta-analysis of 203 studies by *Fox et al*. found the prevalence of TB disease in close contacts was 3.1% in 95 studies in low- and middle-income countries, with a TB prevalence of 1.4% in 108 studies in high-income countries [4]. Another study by *Fox et al*. found that active case finding was more effective than passive case finding alone in Vietnam, a high-prevalence country, and during their investigation noted a TB incidence of 1.13% among close contacts [5]. A recent study by *Krishnan et al*. following household close contacts of rifampin- and multidrug-resistant TB found the cumulative 1-year incidence of TB disease to be 2.3% among the assessed household contacts [6].

Our study aims to supplement this growing body of research and determine the timeline and risk factors for the progression from exposure to active TB in a high-risk population. Prior studies have predominantly been conducted in high-prevalence settings, focused on household contacts, or have been retrospective in nature [3–6]. In this study, we describe pulmonary TB disease progression within a large, national prospective cohort of index patients and all known close contacts in a country with low TB burden but high rates of drug-resistant TB. We have previously described the QuantiFERON-TB Gold Plus seroconversion rates, reflecting TB infection, among a subset of this cohort [7]. We now expand upon this analysis to describe the cohort in detail, identify the rates of progression to TB disease, and identify risk factors for the progression of TB disease. By identifying key factors and timelines associated with the development of active TB, our findings could inform targeted interventions, improve prognostic tools, and ultimately contribute to the global effort in TB control and prevention.

## Materials and methods

This study aimed to determine the incidence of TB disease among close contacts of patients with newly diagnosed TB. Initial recruitment involved identifying participants with newly diagnosed TB, and these individuals were designated as index cases. Subsequently, index case participants were asked to identify close contacts to the best of their ability and recollection. These close contacts were monitored over a period of at least 24 months to determine rates of progression to TB disease. Demographic information, exposure history, clinical data, and clinical blood samples were collected from both index participants and close contacts with the goals of confirming TB disease status and identifying risk factors for progression among close contacts. Clinical blood samples were also collected with the secondary aim of use in potential future studies to evaluate transmission dynamics, risk factors, and TB diagnosis.

This study was conducted in collaboration with the Republic of Moldova’s National TB Program (NTP), encompassing multiple TB clinics, a contact tracing program, and the National TB Reference Laboratory (NTRL). This study was approved by the Ethics Committee of the Institute of Phthisiopneumology "Chiril Draganiuc" in the Republic of Moldova, the University of California San Diego Human Research Protections Program (Project #180068), the University of Arkansas Institutional Review Board (Protocol #1806126401), and the US Department of Defense Office of Human Research Oversight (Project #E00212.1a). Study staff obtained written informed consent in Romanian from all recruited participants, and consent forms were stored securely by the study team in the Republic of Moldova. When applicable, consent was obtained from minors’ parents or guardians.

### Participant enrollment

Participants were recruited from October 1, 2018, through December 31, 2021. Recruitment occurred throughout the Republic of Moldova with the exclusion of Transnistria. Screening for index cases was initiated through clinic microscopy centers and family doctors caring for referred patients. The national online TB registry, the System of Information for Monitoring and Evaluation of TB patients (SIME-TB), was also monitored for new TB diagnoses for potential recruitment. Potential index case participants were approached, screened, and were invited to participate in the study if eligible. Index cases were eligible to participate if they were aged 15 years or older, confirmed positive by smear (grade 1+ or greater) or GeneXpert MTB/RIF (Cepheid, Sunnyvale, CA, USA) within the past four weeks, had not been on tuberculosis treatment for greater than one month, were not currently incarcerated, were willing to provide blood samples, and were willing to provide information on close contacts.

Study nurses and staff involved in recruitment efforts coordinated with the Moldovan National TB Program staff to identify close contacts of recruited index cases. Close contacts were then approached, screened, and, if eligible, invited to participate in the study. Close contacts were considered eligible if they were aged five or older and weighed at least 16 kg, intended to remain in Moldova for 24 months or be available for face-to-face interviews at required follow-up time points, self-reported no prior TB infection, self-reported not having been treated to prevent tuberculosis, were not pregnant, were not currently incarcerated, and were willing to provide blood samples.

Eligibility criteria were relaxed in 2020 to increase recruitment of close contacts, and potential index participants who did not want to provide blood samples became eligible for inclusion. Considering challenges tied to participant mobility and health system burdens, which were increasingly experienced during the COVID-19 pandemic beginning in 2020 and in the context of regional geopolitical pressures in adjacent Ukraine in 2022, participants who were unable to participate in study visits at planned times were seen when feasible by the study team or their clinical status evaluated in coordination with their local family doctor. Following enrollment, all participants could elect at any time to decline to answer one or more questionnaire items, decline to provide one or more blood samples at follow-up, or choose to withdraw from the study.

### Data and specimen handling

The Moldova study team performed all recruitment, questionnaire administration, and sample collection. All data and samples were de-identified in Moldova. Subsequently, de-identified data were securely transferred to the data team at the University of Arkansas, Fayetteville, and biological samples transferred to the University of California, San Diego team and collaborators. All case report forms were stored on a secured REDCap server, and additional clinical data were stored on a secured study database.

### Clinical data collection

Questionnaires collecting demographic information, medical history, travel history, and exposure history were translated into Romanian and administered to all index cases and close contacts at baseline. Clinical data relevant to designating TB infection or disease status were collected at baseline for index cases and close contacts, as well as at follow-up visits for close contacts. Follow-up interviews with close contacts were planned at 3 months, 6 months, 12 months, and 24 months following recruitment. Relevant clinical outcome data were collected at these times to identify individuals who had progressed to TB disease or had experienced relevant changes in their medical history. Due to logistical restraints related to the COVID-19 pandemic, additional in-person study visits at 24 months were eliminated in 2020. However, additional sources were used to supplement primary data collection throughout the study period, including microbiological records from the central TB laboratory in Chisinau and clinical data collected by regularly reviewing the online national TB registry (SIME-TB). This approach was used to identify participants who had progressed to TB disease outside of the scheduled in-person interview periods and to capture relevant clinical data not obtained during regular study visits due to visit timing or logistical constraints. These registries were continuously monitored and reviewed at least quarterly for data pertaining to enrolled participants. All close contacts were routinely reviewed for TB disease based on clinical evaluation by the participant’s primary treating physician, Moldova team physicians, and study physicians at the University of California, San Diego. Close contacts who did not develop TB disease over the course of the study were designated as “non-progressor” cases. Close contacts with evidence of TB disease identified within 30 days of index case treatment initiation were considered “co-prevalent” cases given temporal proximity to the index case infectious period. Close contacts who had no evidence of clinical TB disease on recruitment and developed TB disease 30 days or more after index case treatment initiation were considered “progressor” cases.

### Blood sample collection

Blood samples were collected from eligible participants for concurrent and planned studies [7]. Participants who agreed to provide blood samples had 30 mL of blood drawn into EDTA tubes, collected on-site during patient visits and processed at the central lab in Chisinau. PBMCs were purified from whole blood by Ficoll gradient centrifugation and stored in Falcon (Becton, Dickinson and Company, Franklin Lakes, NJ, USA) and SepMate (STEMCELL Technologies Inc., Vancouver, Canada) tubes. Plasma samples were stored at -80°C, and PBMCs were stored on liquid nitrogen. An additional 2.5 mL of blood was drawn and used for stabilization of intracellular RNA using PAXgene Blood RNA Tubes (Becton, Dickinson and Company, Franklin Lakes, NJ, USA) and stored at -80°C, and 4mL of blood were used for testing with QuantiFERON–TB Gold Plus (Qiagen, Hilden, Germany). A subset of participants also had 3mL of blood drawn for testing by LIOFeron-TB/LTBI (Lionex Diagnostics & Therapeutics GmbH, Braunschweig, Germany). Testing for HIV status, CD4 T-cell count, and glycated hemoglobin A1c were also performed at the time of recruitment; however, participants could decline HIV and CD4 testing.

### Statistical analysis

Means were compared using the Mann-Whitney U-test, proportions were compared using the z-test, and categorical variables were evaluated using Fisher’s exact test. Regression analysis was done using logistic regression. Statistical analyses were conducted using Stata 17 (StataCorp LLC, College Station, TX, USA).

## Results

### Participant recruitment

Of 421 potential index cases screened, 285 met eligibility criteria and consented to participate in the study. Sixty index case participants declined to have blood drawn for the purposes of the study but agreed to provide clinical data and identify close contacts. Index case participants identified 1143 close contacts, of whom 1120 met eligibility criteria and consented to participate in the study. Two close contacts declined to have blood drawn and were included in the study but did not have blood laboratory data collected, and 138 close contacts withdrew from the study over the study period. A high degree of variability in observed follow-up visit frequency and timing occurred among close contacts despite efforts by the research team. Of the 982 close contacts assessed at baseline and included in the final analysis, 708 participants were seen for their 6-month study visit, and 278 were seen for their 12-month study visit. However, many participants followed up off-schedule with 850 close contacts evaluated at least once after their baseline visit. Additionally, relevant data for 58 close contacts were identified through the national TB registry (SIME-TB), with regular review of the registry performed through November 8, 2022, and supplementary data on identified active cases collected through July 31, 2023.

### Participant demographics, risk factors, and exposures

Demographic characteristics for index participants are given in Table 1. Index participants were predominantly men (77.1%) with a median age of 43 (interquartile range [IQR] 35–54). The median weight of index participants was 59.0 kg (IQR 52.0–65.0 kg), with a corresponding median body mass index (BMI) of 20.2 (IQR 18.5–22.1 kg/m^2^). Nine participants were living with HIV (4.7%). Other comorbidities reported by index participants included diabetes (9/285), hepatitis B (4/285), hepatitis C (2/285), asthma (1/285), chronic kidney disease (1/285), and immunosuppressive conditions (1/285). Among index participants with diabetes, the median hemoglobin A1c was 9.2% (IQR 7.6–18.0%). The majority of index participants reported active tobacco use (63.0%). Three index participants declined to identify their country of birth, and of the remaining 282 participants, 279 (98.9%) reported being born in Moldova, and three were born in Ukraine (1.1%). Of the 40 index participants who traveled outside of Moldova within the previous 12 months, 39 reported having been born in Moldova, and one participant did not respond to the question. Three index participants reported planned travel outside the country in the next 12 months following recruitment, with one planning to travel to France and two planning to travel to Russia. The median household size was two members (IQR 2–3.5 members), and index participants reported regular contact with a median of one person outside the household (IQR 0–3 persons).

**Table 1:** Characteristics of index cases.

Characteristic	Index Participants
**Male sex**–n/N (%)	219/284 (77.1%)
**Age**–years	
15–29	32/283 (11.3%)
30–44	121/283 (42.8%)
45–59	97/283 (34.3%)
60–64	31/283 (11.0%)
75+	2/283 (0.7%)
**Living with HIV**–n/N (%)	9/193 (4.7%)
**Prior TB infection–n/N (%)**	35/280 (12.5%)
**Current tobacco smoker**–n/N (%)	175/278 (63.0%)
**Country of birth**–n/N (%)	
Moldova	279/282 (98.9%)
Russia	0/282 (0.0%)
Georgia	0/282 (0.0%)
Ukraine	3/282 (1.1%)
Other	0/282 (0.0%)
**Traveled outside Moldova within 12 months**–n/N (%)	
Russia	28/284 (9.9%)
Georgia	0/284 (0.0%)
Other	12/284 (4.2%)
**Employment status**–n/N (%)	
Full-time	42/283 (14.8%)
Part-time	9/283 (3.2%)
Unemployed	199/283 (70.3%)
Retired	21/283 (7.4%)
Student	1/283 (0.4%)
Disabled	11/283 (3.9%)
**Household type**–n/N (%)	
Independent housing	261/285 (91.6%)
Group housing	1/285 (0.4%)
Unhoused	0/285 (0.0%)
**Household size (including participant)**–n/N (%)	
1	33/144 (22.9%)
2	41/144 (28.5%)
3–4	57/144 (39.6%)
5–7	9/144 (6.3%)
8–10	2/144 (1.4%)
11+	2/144 (1.4%)
**Number of people outside the household with whom you have regular contact**–n/N (%)	
0	83/173 (48.0%)
1	28/173 (16.2%)
2–3	37/173 (21.4%)
4–5	11/173 (6.4%)
6–8	4/173 (2.3%)
9–11	6/173 (3.5%)
12–14	2/173 (1.2%)
15+	2/173 (1.2%)

The number of respondents for each item may be fewer than the number of participants interviewed, as participants could decline to answer interview questions or provide demographic information for any item.

Of the 982 close contacts included in the final analysis, 23 (2.3%) were identified as progressor cases, defined as having a diagnosis date more than 30 days after the date of index case treatment initiation, and 13 (1.3%) were identified as co-prevalent cases, defined as having a diagnosis date within 30 days of the treatment initiation date of their index case. Among the progressors, two participants developed TB disease within three months of recruitment, eight developed TB disease between three months and one year following recruitment, 11 developed TB disease between one year and two years after recruitment, and two developed TB disease more than two years after recruitment. The total incidence rate of TB disease among all close contacts was 3.7% (36/982). Baseline characteristics for close contact participants are given in Table 2. In contrast to the index population, close contact participants were predominantly women (57.2%). The median age of all close contacts was 44 (IQR 31–58). The median weight among non-progressor participants was 69.0 kg (IQR 59.0–80.0 kg), compared to 67.0 kg (IQR 52.0–73.0 kg) among progressors and 65.0 kg (IQR 52.0–73.0 kg) among pooled progressor and co-prevalent cases. The median BMI was 24.8 kg/m^2^ (IQR 59.0–80.0 kg/m^2^) among non-progressors, 22.6 kg/m^2^ (IQR 21.4–25.5 kg/m^2^) among progressors, and 23.2 kg/m^2^ (IQR 21.0–25.5 kg/m^2^) among pooled progressor and co-prevalent cases. The median weight was not significantly different between non-progressors and progressors (p = 0.116) at the α = 0.05 level; however, the median BMI between these groups was significantly different (p = 0.025). Nine (0.9%) close contacts reported a diagnosis of HIV, among whom none developed TB disease over the course of the study. All close contacts living with HIV declined additional CD4 testing. Other comorbidities among close contacts included diabetes (31/982), hepatitis B (9/982), hepatitis C (7/982), asthma (5/982), and chronic kidney disease (3/982). Among close contacts with diabetes, the median hemoglobin A1c was 7.9% (IQR 6.9–9.3%). Tobacco smoking was reported by 21.8% among non-progressors, compared to 50.0% among close contacts diagnosed with TB disease. Almost all close contacts had a history of BCG vaccination with scar identified (98.65%) and reported being born in Moldova (99.8%). Among close contacts who developed TB disease over the course of the study, none had traveled outside of the country within the previous 12 months.

**Table 2:** Characteristics of close contacts.

Characteristic	Non-progressors	Progressors		Any TB Disease (Progressor or Co-prevalent)	
			p-value		p-value
**Male sex**–n/N (%)	397/944 (42.1%)	12/23 (52.2%)	0.332	22/36 (61.1%)	**0.023**
**Age**–years, n/N (%)			0.158*		0.072*
5–14	69/943 (7.3%)	3/23 (13.0%)		3/36 (8.3%)	
15–29	152/943 (16.1%)	3/23 (13.0%)		5/36 (13.9%)	
30–44	267/943 (28.3%)	11/23 (47.8%)		18/36 (50.0%)	
45–59	259/943 (27.5%)	5/23 (21.7%)		8/36 (22.2%)	
60–75	188/943 (19.9%)	1/23 (4.4%)		2/36 (5.6%)	
75+	8/943 (0.9%)	0/23 (0.0%)		0/36 (0.0%)	
**Weight**–kg, n/N (%)			0.268*		0.096*
<20	1/945 (0.1%)	0/23 (0.0%)		0/36 (0.0%)	
20–39	47/945 (5.0%)	0/23 (0.0%)		0/36 (0.0%)	
40–59	189/945 (20.0%)	9/23 (39.1%)		13/36 (36.1%)	
60–79	441/945 (46.7%)	12/23 (52.2%)		20/36 (55.6%)	
80–99	223/945 (23.60%)	2/23 (8.7%)		3/36 (8.3%)	
100–119	31/945 (3.3%)	0/23 (0.0%)		0/36 (0.0%)	
120–139	10/945 (1.1%)	0/23 (0.0%)		0/36 (0.0%)	
>140	3/945 (0.3%)	0/23 (0.0%)		0/36 (0.0%)	
**BMI**–kg/m^2^, n/N (%)			0.688*		0.236*
<16.0	20/944 (2.1%)	0/23 (0.0%)		0/36 (0.0%)	
16.0–18.4	49/944 (5.2%)	2/23 (8.7%)		4/36 (11.1%)	
18.5–24.9	427/944 (45.2%)	14/23 (60.9%)		20/36 (55.6%)	
25.0–29.9	270/944 (28.6%)	6/23 (26.1%)		11/36 (30.6%)	
30.0–34.9	126/944 (13.4%)	1/23 (4.4%)		1/36 (2.8%)	
35.0–39.9	33/944 (3.5%)	0/23 (0.0%)		0/36 (0.0%)	
>40.0	19/944 (2.0%)	0/23 (0.0%)		0/36 (0.0%)	
**Living with HIV**–n/N (%)	9/931 (1.0%)	0/22 (0.0%)	0.646	0/34 (0.0%)	0.562
**Prior TB infection–n/N (%)**	3/804 (0.4%)	0/18 (0.0%)	0.795	1/28 (3.6%)	**0.016**
**Current tobacco smoker**–n/N (%)	204/937 (21.8%)	10/23 (43.5%)	**0.014**	17/34 (50.0%)	**<0.001**
**Country of birth**–n/N (%)			>0.999*		>0.999*
Moldova	931/933 (99.8%)	23/23 (100.0%)		36/36 (100.0%)	
Russia	0/933 (0.0%)	0/23 (0.0%)		0/38 (0.0%)	
Ukraine	1/933 (0.1%)	0/23 (0.0%)		0/38 (0.0%)	
Other	1/933 (0.1%)	0/23 (0.0%)		0/38 (0.0%)	
**Traveled outside Moldova within 12 months**–n/N (%)			>0.999*		0.779*
Russia	14/938 (1.5%)	0/23 (0.0%)		0/36 (0.0%)	
Georgia	0/938 (0.0%)	0/23 (0.0%)		0/36 (0.0%)	
Other country	30/938 (3.2%)	0/23 (0.0%)		0/36 (0.0%)	
No travel	894/938 (95.3%)	23/23 (100.0%)		36/36 (100.0%)	
**Employment status**–n/N (%)			**0.050** *		**0.006** *
Full-time	199/924 (21.5%)	3/23 (13.0%)		6/36 (16.7%)	
Part-time	35/924 (3.8%)	2/23 (8.7%)		2/36 (5.6%)	
Unemployed	409/924 (44.3%)	13/23 (56.5%)		19/36 (52.8%)	
Retired	156/924 (16.9%)	0/23 (0.0%)		0/36 (0.0%)	
Student	80/924 (8.7%)	3/23 (13.0%)		4/36 (11.1%)	
Disabled	45/924 (4.9%)	2/23 (8.7%)		5/36 (13.9%)	

The number of respondents for each item may be fewer than the number of participants interviewed, as participants could decline to answer interview questions or provide demographic information for any item. P-values derived from Fisher’s exact test are indicated with

*, and all other p-values represent z-test values. Bolded p-values indicate values less than or equal to 0.05.

**Fig 1:**
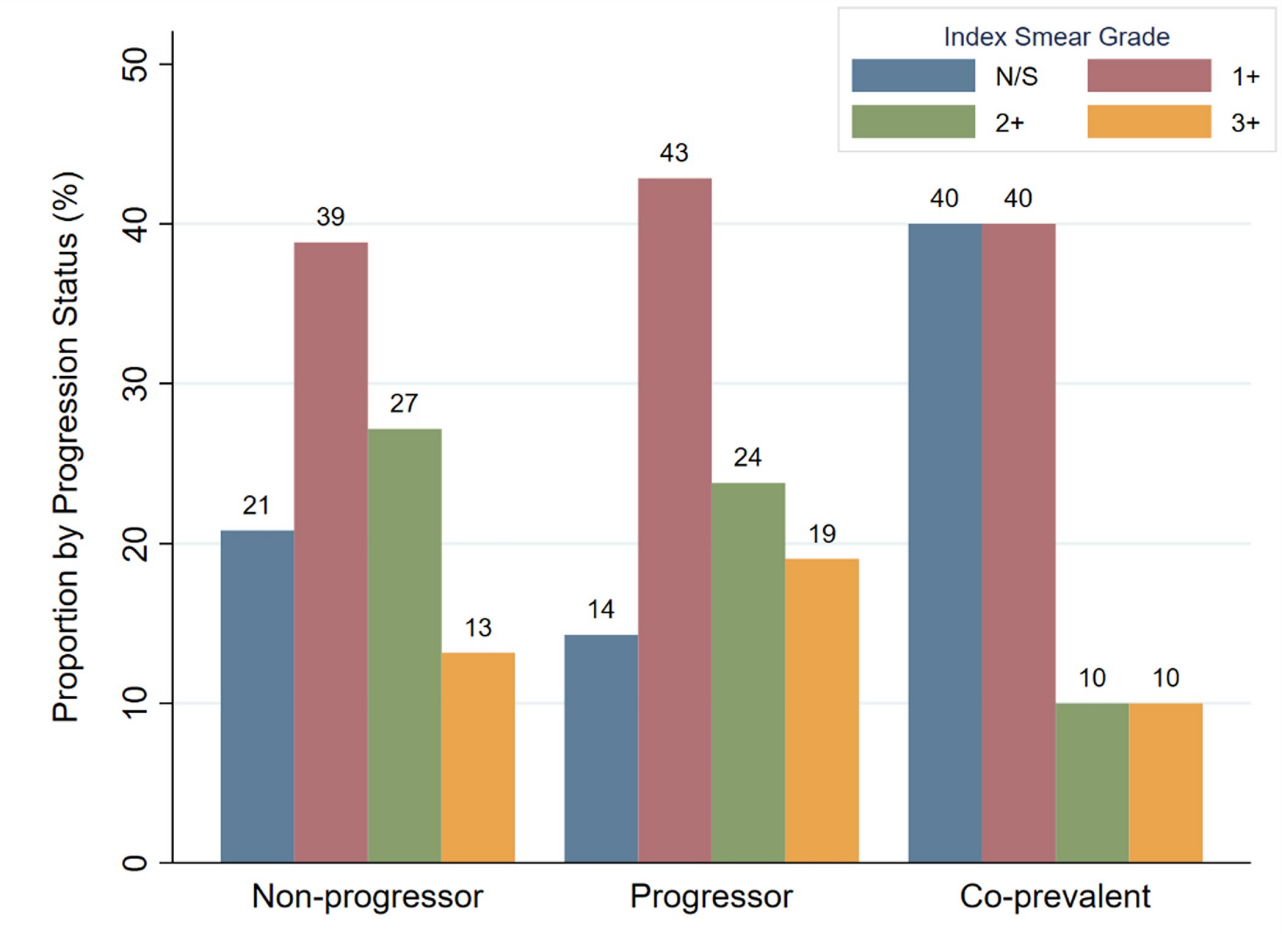
Close contact TB disease status by index AFB smear grade exposure. The proportions of index participant baseline AFB smear grades are shown by progression status. “N/S” denotes a negative or scanty smear grade. Close contacts for whom the corresponding index AFB smear status was not available have been omitted (non-progressor n = 744, progressor n = 21, co-prevalent n = 10). Progression to TB disease among close contacts, either with progressor or co-prevalent status, was not found to be related to their index participant’s baseline AFB smear grades (Fisher’s exact p = 0.791).

**Fig 2:**
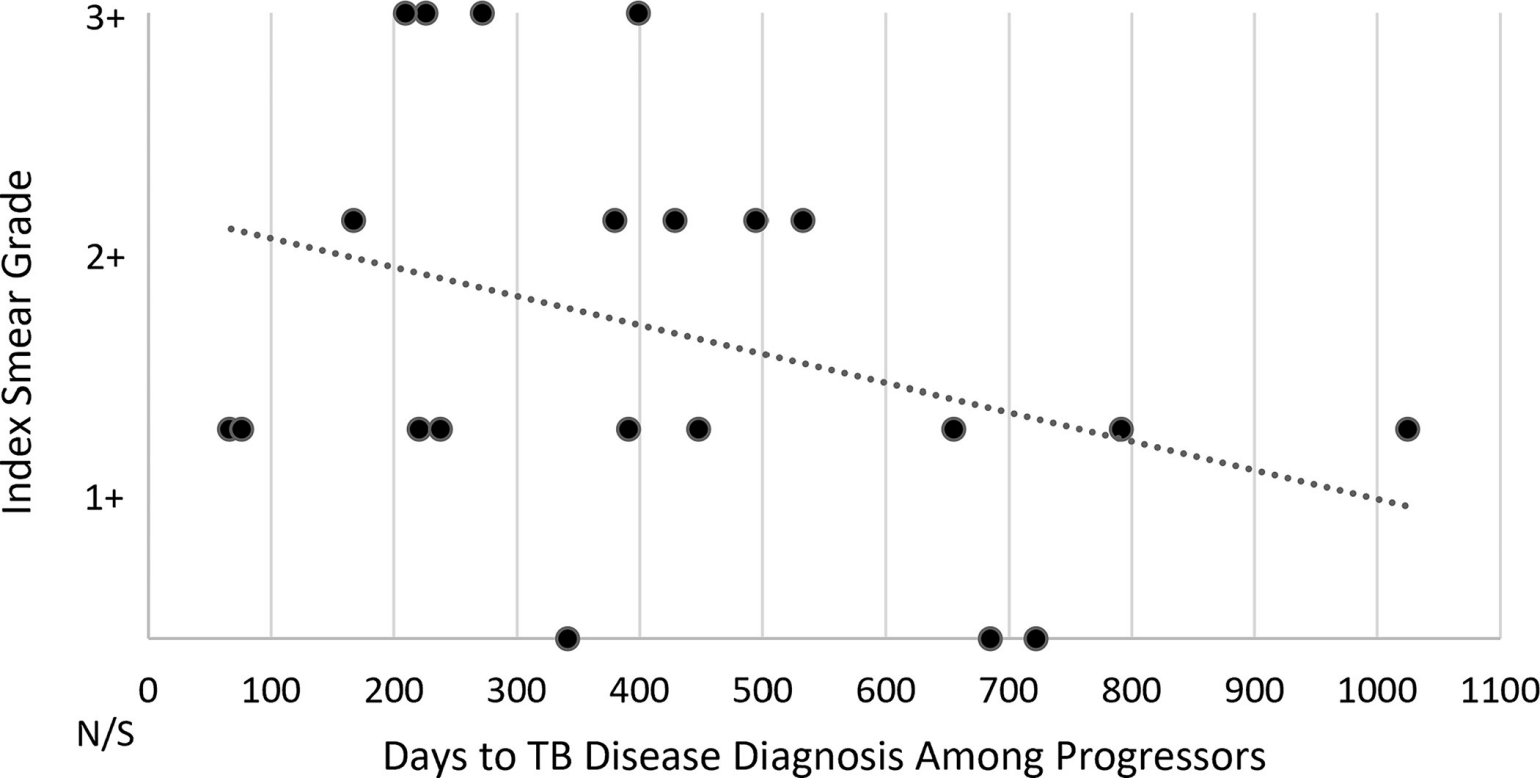
Progressor close contact days to TB disease by index baseline smear grade. The number of days from the end of index participants’ infectious periods to the diagnosis of TB disease among progressor close contacts is shown above by index participant baseline smear grade, with each black dot representing a progressor close contact. “N/S” denotes a negative or scanty smear grade. Progressors for whom index AFB smear status was not available have been omitted. Assigning smear grade to a linear scale from zero to three, with negative and scanty smears graded as zero, produces a linear correlation with r = –0.351 and r^2^ = 0.123, shown above as a dotted line.

Exposure setting, frequency, and duration of close contacts to index participants are given in Table 3. Most close contacts reported exposure to an index participant in an independent household setting (88.3%), while smaller numbers reported exposure in a group household (1.1%), workplace (10.1%), place of worship (0.5%), bar or club (1.1%), or other location (1.6%). Progressors and co-prevalent cases had higher exposure frequency and duration to index individuals than non-progressors. When comparing daily exposure to less than daily exposure, progressors were more likely to have daily contact with the index individual than non-progressors (Fisher’s exact p = 0.036). When controlling for age, BMI, and tobacco use, close contacts who reported daily contact of more than eight hours with an index participant had an odds ratio (OR) of 4.28 (p = 0.001, 95% CI 1.79–10.23) for being a progressor, and an OR of 4.08 (p<0.001, 95% CI 1.98–8.38) for being a progressor or co-prevalent, when compared to close contacts who had exposures that were less frequent or of shorter durations (Table 4). The inclusion of employment status as an additional control variable in the model was also evaluated as a robustness analysis and produced an OR of 4.61 (p = 0.001, 95% CI 1.86–11.43) for being a progressor and an OR of 4.34 (p<0.001, 95% CI 2.05–9.21) for being a progressor or co-prevalent. The development of TB disease among close contacts was not found to be related to the AFB smear grade of their corresponding index cases in the cohort (Fig 1). Among progressor close contacts, the number of days to develop TB disease trended towards an inverse relationship with index baseline smear grade (linear correlation with r = –0.351 and r^2^ = 0.123, Fig 2).

**Table 3:** Close contact exposures to index participants.

Characteristic	Non-progressors	Progressors		Any TB Disease (Progressor or Co-prevalent)	
			p-value		p-value
Any household contact with index case, by type–n/N (%)			0.269*		0.390*
Independent household	828/940 (88.1%)	21/23 (91.3%)		34/36 (94.4%)	
Group household	10/940 (1.1%)	1/23 (4.3%)		1/36 (2.8%)	
Unhoused	1/940 (0.1%)	0/23 (0.0%)		0/36 (0.0%)	
Any non-household contact with index case, by location–n/N (%)			>0.999*		>0.999*
Workplace	97/940 (10.3%)	1/23 (4.3%)		2/36 (5.6%)	
School	0/940 (0.0%)	0/23 (0.0%)		0/36 (0.0%)	
Childcare center	0/940 (0.0%)	0/23 (0.0%)		0/36 (0.0%)	
Place of worship	5/940 (0.5%)	0/23 (0.0%)		0/36 (0.0%)	
Community center	0/940 (0.0%)	0/23 (0.0%)		0/36 (0.0%)	
Volunteer setting	0/940 (0.0%)	0/23 (0.0%)		0/36 (0.0%)	
Bar/club	11/940 (1.2%)	0/23 (0.0%)		0/36 (0.0%)	
Other	15/940 (1.6%)	0/23 (0.0%)		0/36 (0.0%)	
Average frequency of contact with index case–n/N (%)			0.330*		0.119*
Daily	442/939 (47.1%)	16/23 (69.6%)		25/34 (73.5%)	
4 to 6 times per week	180/939 (19.2%)	2/23 (8.7%)		2/34 (5.9%)	
2 to 3 times per week	187/939 (19.9%)	4/23 (17.4%)		5/34 (14.7%)	
Once per week	88/939 (9.4%)	0/23 (0.0%)		1/34 (2.9%)	
2 to 3 times per month	30/939 (3.2%)	1/23 (4.4%)		1/34 (2.9%)	
Once per month	6/939 (0.6%)	0/23 (0.0%)		0/34 (0.0%)	
Less than once per month	6/939 (0.6%)	0/23 (0.0%)		0/34 (0.0%)	
Average duration of contact with index case–n/N (%)			**0.031** *		**0.004** *
>8 hours	280/939 (29.8%)	14/23 (60.9%)		20/34 (58.8%)	
4 to 8 hours	249/939 (26.5%)	4/23 (17.4%)		8/34 (23.5%)	
2 to <4 hours	256/939 (27.3%)	5/23 (21.7%)		6/34 (17.7%)	
1 to <2 hours	123/939 (13.1%)	0/23 (0.0%)		0/34 (0.0%)	
Less than 1 hour	31/939 (3.3%)	0/23 (0.0%)		0/34 (0.0%)	
Lived or worked with person with TB other than index case–n/N (%)	133/939 (14.6%)	3/23 (13.0%)	0.881	3/34 (8.8%)	0.379

The number of respondents for each item may be fewer than the number of participants interviewed, as participants could decline to answer interview questions or provide demographic information for any item. P-values derived from Fisher’s exact test are indicated with

*, and all other p-values represent z-test values. Bolded p-values indicate values less than or equal to 0.05.

**Table 4:** Odds of developing TB disease by exposure to index participant.

Contact Exposure to Index Participant	Progressor		Any TB Disease (Progressor or Co-prevalent)	
	OR (95% CI)	Adjusted OR (95% CI)*	OR (95% CI)	Adjusted OR (95% CI)*
Less than daily or less than 8 hours	1.00	1.00	1.00	1.00
Daily and for more than 8 hours	4.41 (1.88–10.31)	4.28 (1.79–10.23)	4.05 (2.01–8.14)	4.08 (1.98–8.38)

Close contact odds of developing TB disease by high exposure frequency and duration, defined as daily and for more than eight hours, compared to a reference of non-high exposure, defined as less than daily contact or for durations of less than eight hours. Odds ratios are given for the outcomes of progressor status and any TB disease status (progressor or co-prevalent).

* Adjusted for age, BMI, and active tobacco use.

## Discussion

Our study followed a large cohort of participants with pulmonary TB disease and their close contacts over 58 months and ultimately identified a pulmonary TB disease incidence rate of 3.7% among all close contacts and 2.3% among close contacts identified as progressors. These incidence rates are in line with recently reported rates among close contacts, as described previously [3–6]. Close contacts in the cohort who experienced longer and more frequent contact with index cases were at increased risk for developing TB disease, and is consistent with prior studies that have demonstrated increased exposure to pulmonary TB increases the risk for TB infection and disease [8]. Identified risk factors for TB disease among close contacts included male sex, active tobacco use, prior TB infection, and employment status. Official labor participation rates in Moldova have been reported between 42–46% from 2022–2023, with many falling outside the formal labor economy and engaging in informal work [9]. Higher rates of unemployment were observed among close contacts who developed TB disease and likely reflect the consequences of lower socioeconomic status, serving as a proxy for TB risk factors such as crowding, decreased ventilation, and indoor solid fuel use in both household and non-household settings. Index sputum smear grade was not found to predict progression to disease among close contacts, although higher smear grades were found to have a weak association with shorter time to active disease among those who did progress. This finding contrasts with other studies that demonstrate that higher sputum AFB smear grades are associated with an increased risk of TB transmission [10]. This discrepancy likely reflects the low number of progressors observed in our studies limiting statistical evaluation, as the study was not designed to investigate this outcome. However, it is notable that among close contacts who developed TB disease, 22.4% had corresponding index negative or scanty smear grades.

Recruited index participants were predominantly men (77.1%), which may reflect biological differences in TB infection or inequity in health care access by sex, among other potential factors. This increased male-to-female ratio has been observed in multiple prior TB prevalence surveys [11]. Notably, while the proportion of women was higher among close contacts (57.2%) than index participants, close contacts that developed TB disease were predominantly men (61.1%). As many index participants reported their domestic partners as close contacts, the higher proportion of men initially recruited as index participants likely led to increased recruitment of women among close contacts. This tendency may have been further accentuated by the fact that most close contacts recruited were found to have occurred in the household setting, with fewer close contacts reported from other potential exposure settings such as school, work, places of worship, or recreational spaces.

We should note that although close contacts from various exposure settings were sought in this study, 91.7% of close contacts reported that their exposure to the index participant occurred in a household setting. This high proportion of close contact exposure in the household is higher than is commonly cited in the literature, with prior studies reporting 51–85% of close contacts occurring in the household in the high-prevalence settings of Zambia, South Africa, and Vietnam, and 18–32% in eight European countries [12–14]. A simulation parameterized using empirical data from Cape Town, South Africa estimated that meetings between household members accounted for 36% of adult meeting time and household transmissions accounted for approximately 21% of transmitted TB disease [15]. Similarly, molecular analyses have estimated that only 8–19% of tuberculosis cases were attributable to household transmission in two studies in South Africa [16, 17]. Our recruited population, therefore, likely overrepresents close contacts from the household.

This study has multiple strengths. Recruitment was widely representative of the Moldovan population, with recruitment occurring at multiple TB centers in multiple regions and drawing from cities and villages across the country. High baseline participation rates were observed among eligible individuals, and a long monitoring period supplemented by the national SIME-TB surveillance system allowed for extensive clinical data during and beyond the designated in-person clinical follow-up period. The limitations of this study include a smaller than anticipated sample size related to challenges experienced during the recruitment of close contacts due to disruptions related to the COVID-19 pandemic. The timings of follow-up visits were also less consistent as a result of strains on the health care system and in the setting of recommended decreased social contact during this period. Additionally, the recruitment of high-risk populations, including temporary residents and migrants, among others, may have been impacted as non-critical health and social services engaging these populations faced similar challenges during the pandemic. Of the recruited index participants, 4.7% had a diagnosis of HIV, compared to 0.9% among recruited close contacts. This sample size limited subgroup analysis among people living with HIV; however, the observed prevalence is consistent with the Moldova general population HIV prevalence estimate of 0.3% [18].

## Conclusions

In this close contacts study in the Republic of Moldova, the incidence of TB disease following exposure was 3.7% among all close contacts. While our findings of TB disease incidence among close contacts and risk factors for progression should be considered in the context of Moldova, they align with published literature from various countries and contexts. This observed consistency across multiple studies conducted globally suggests a “universal” progression rate ranging from 1–4% among close contacts and is likely related to *Mycobacterium tuberculosis* biology and TB disease pathogenesis.

Current public health methods for TB infection and disease management commonly involve contact tracing for testing and, if appropriate, treatment. Recent publications have suggested incorporating approaches such as active case finding in hard-to-reach populations [19]. However, given the difficulty of predicting who will ultimately develop TB disease and the challenges inherent in established methods such as contact tracing, individual patient and population-level management of TB will require enhanced tools to improve diagnosis and appropriate treatments. A future step towards achieving this goal would be to use clinical data and samples collected in this and similar studies to develop a better understanding of timelines and risk factors in specific populations and more precise and effective biomarkers to identify individuals at the highest risk for progression. Overall, our study and similar studies will allow us to better understand and characterize the entire spectrum of TB from exposure to infection, disease, and recovery.

## Supporting information

S1 File Inclusivity in global research. (DOCX)
