# Supplementary material for: Understanding tuberculosis transmission and progression: A prospective cohort study of index cases and close contacts in Moldova
Source: PLoS One. 2024 Dec 5;19(12):e0313270. doi: 10.1371/journal.pone.0313270 (PMC11620447; doi:10.1371/journal.pone.0313270)
Supplement: S1 File — (DOCX) [file pone.0313270.s001.docx]

Inclusivity in global research

PLOS’ policy on inclusivity in global research aims to improve transparency in the reporting of research performed outside of researchers’ own country or community and ensures that PLOS publications reporting global research adhere to high standards for research ethics and authorship. Authors of relevant research articles may be asked to complete the questionnaire below, which outlines ethical, cultural, and scientific considerations specific to inclusivity in global research. This questionnaire may be requested when researchers have travelled to a different country to conduct research, if research uses samples collected in another country, research with Indigenous populations or their lands, or if research is on cultural artefacts. Researchers travelling to another country solely to use laboratory equipment will not normally be required to complete the questionnaire. However, the questionnaire can be requested at the journal’s discretion for any submission – if you have been requested to complete this questionnaire by the PLOS journal you submitted to, please do so.

Please complete the questionnaire below and include this as a Supporting Information file with your manuscript. Note that if your paper is accepted for publication, this checklist will be published with your article in the supporting information files. Please ensure that you reference the checklist in the main body of your manuscript. We suggest adding a subsection ‘Inclusivity in global research’ to your Methods section and adding the following sentence: “Additional information regarding the ethical, cultural, and scientific considerations specific to inclusivity in global research is included in the Supporting Information (SX Checklist)”

The questions have been designed to be applicable to a wide range of study types, and there are subsections for both human subjects research and non-human subjects research. If any of the questions are not relevant to your research please mark them as “N/A” as appropriate.

**Ethical considerations, permits and authorship**

*This section is applicable to all research types.*

Provide details as to who granted permissions and/or consent for the study to take place in the Methods section of your manuscript. This should include the names of **all** ethics boards, governmental organizations, community leaders or other bodies that provided approval for the study. If individuals provided approval refer to these people by their role or title but do not list their name(s).

Reported on page number: 6 of the manuscript (with tracked changes)

If there were any deviations from the study protocol after approval was obtained please provide details of these changes in the Methods section of your manuscript.
Did this study involve local collaborators that are residents of the country where the research was conducted or members of the community studied? If you do not have any authors from said communities, please provide an explanation for this below.

Yes, this work was conducted as a collaborate project and includes multiple authors from the Republic of Moldova. Specifically, authors Valeriu Crudu, Elena Tudor, Nelly Ciobanu, and Alexandru Codreanu are members of the Institute of Phthisiopneumology “Chiril Draganiuc” in Chisinau, Republic of Moldova.

Reported on page number: There were no deviations from the approved protocols. In the context of the COVID-19 pandemic, index participants could decline to provide blood samples and in person 24-month visits among close contacts were replaced by data from the national TB surveillance database. This is detailed on pages 8 and 9 of the manuscript (with tracked changes).

Everyone listed as an author should meet PLOS’ criteria for authorship and all individuals who meet these criteria should be included in the author byline, rather than the acknowledgements. For further information please see the journal’s Authorship Policy.

**Human subjects research (e.g. health research, medical research, cross-cultural psychology)**

Did you obtain written informed consent from a representative of the local community or region before the research took place? How did you establish who speaks for the community? Details of written informed consent obtained from study participants should be reported separately in the Methods section of your manuscript.

As our research aimed to investigated TB incidence at the national level in the Republic of Moldova, the work was conducted closely with the Republic of Moldova’s National TB Program. Study authors Valeriu Crudu, Elena Tudor, Nelly Ciobanu, and Alexandru Codreanu are key members of the National TB Program, and represent multiple areas of their work including clinical assessments, microbiologic laboratory data, contact tracing, and program implenetation, among others. All participants in the study provided written informed consent. All consent procedures were conducted in Romanian, the official language of the Republic of Moldova. This is noted in the Methods section on page 6 of the manuscript (with tracked changes).

How did members of the local community provide input on the aims of the research investigation, its methodology, and its anticipated outcome(s)?

The research design and implementation were developed and executed with key members of the local community, specifically authors Valeriu Crudu, Elena Tudor, Nelly Ciobanu, and Alexandru Codreanu, as noted previously.

When engaging with the local community, how did you ensure that the informed consent documents and other materials could be understood by local stakeholders?

As mentioned above, all documents and materials were translated and available in English and Romanian. Informed consent procedures were evaluated by multiple individuals, including multiple staff from the National TB Program.

Will the findings of the research be made available in an understandable format to stakeholders in the community where the study was conducted (e.g. via a presentation, summary report, copies of publications, etc.)? Please provide details of how this will be achieved.

Yes, all findings are being made available to the local stakeholders. Given the scope and aims of the project, key stakeholders include the National TB Program and the Institute of Phthisiopneumology “Chiril Draganiuc.” As authors and collaborators, findings and data from the research are shared closely. Sharing of the study findings is being done through correspondence, presentations, data provision, and summary reports. Publication of this manuscript aims to be an additional method of dissemination of the findings from this study.

**Non-human subjects research using specimens/ animals collected as part of the study, or those housed in archival collections. Examples include archaeology, paleontology, botany and zoology.**

Did the permission you obtained from a local authority to perform the study include an agreement on access to outputs and benefit sharing? This may include procedures to enable fair distribution of the benefits and resources arising from the research performed. Please include any details of Prior Informed Consent and Benefit Sharing Agreements obtained. These may be required by field-specific regulations, for example the Convention on Biological Diversity (CBD) and the associated Nagoya Protocol.

If the material used in your study was imported, please A) provide the year it was imported and B) indicate whether permits were obtained to import/export the materials used, C) provide details of any permits obtained. If this information is not available, please indicate this.

If you used archival specimens, please state how the material used in your study was acquired by the institute it is held in and provide details of any permits obtained for the original excavations/ sample collection. If this information is not available, please indicate this.

How was the potential cultural significance of the materials collected in your study to local communities considered in your research design? Were Indigenous peoples and/or local researchers and institutions involved with archaeological excavations / collection of specimens? If so, please provide a description of their involvement.

If your manuscript includes photographs of human remains please indicate whether authors obtained permission from descendants or affiliated cultural communities to do so.
